# Hydroxychloroquine efficacy and safety in preventing SARS-CoV-2 infection and COVID-19 disease severity during pregnancy (COVID-Preg): a structured summary of a study protocol for a randomised placebo controlled trial

**DOI:** 10.1186/s13063-020-04557-y

**Published:** 2020-07-02

**Authors:** Raquel González, Laura García-Otero, Clara Pons-Duran, Elena Marbán-Castro, Anna Goncé, Elisa Llurba, Maria del Mar Gil, Miguel Ángel Rodríguez-Zambrano, Haily Chen, Máximo Ramírez, Azucena Bardají, Clara Menendez

**Affiliations:** 1 ISGlobal, Hospital Clínic-Universitat de Barcelona, Barcelona, Spain; 2 Consorcio de Investigación Biomédica en Red de Epidemiología y Salud Pública (CIBERESP), Barcelona, Spain; 3 Manhiça Health Research Center (CISM), Manhiça, Mozambique; 4 BCNATAL | Barcelona Center for Maternal Fetal and Neonatal Medicine, Hospital Clínic de Barcelona, Barcelona, Spain; 5 Obstetrics and Gynecology Department, Hospital de la Santa Creu i Sant Pau, Barcelona, Spain; 6 Obstetrics and Gynecology Department, Hospital Universitario de Torrejón, Torrejón de Ardoz, Madrid Spain; 7 School of Medicine, Universidad Francisco de Vitoria, Madrid, Spain; 8 HM Puerta del Sur, Móstoles, Madrid Spain

**Keywords:** COVID-19, Randomised controlled trial, Protocol, Hydroxychloroquine, Pregnant, Women

## Abstract

**Objectives:**

The primary objectives of the study are:

1. To assess the effect of hydroxychloroquine (HCQ) in reducing SARS-CoV-2 viral shedding by PCR in infected pregnant women with mild symptoms.

2. To assess the efficacy of HCQ to prevent SARS-CoV-2 infection in pregnant women in contact with an infected or suspected case.

3. To evaluate the effect of HCQ in preventing the development of the COVID-19 disease in asymptomatic SARS-CoV-2-infected pregnant women.

The secondary objectives are:

1. To determine the effect of HCQ on the clinical course and duration of the COVID-19 disease in SARS-CoV-2-infected pregnant women.

2. To determine the impact of HCQ on the risk of hospitalization and mortality of SARS-CoV-2-infected pregnant women.

3. To assess the safety and tolerability of HCQ in pregnant women.

4. To describe the clinical presentation of SARS-CoV-2 infection during pregnancy.

5. To describe the effects of maternal SARS-CoV-2 infection on pregnancy and perinatal outcomes by treatment group.

6. To determine the risk of vertical transmission (intra-utero and intra-partum) of SARS-CoV-2.

**Trial design:**

Randomized double-blind placebo-controlled two-arm multicentre clinical trial to evaluate the safety and efficacy of HCQ to prevent and/or minimize SARS-CoV-2 infection during pregnancy. Participants will be randomized to receive a 14-day oral treatment course of HCQ or placebo, ratio 1:1.

**Participants:**

Study population: pregnant women undergoing routine prenatal follow up or attending emergency units at the participating hospitals who report either symptoms/signs suggestive of COVID-19 disease or close contact with a suspected or confirmed COVID-19 case.

*Inclusion criteria*

Women will be invited to participate in the trial and sign an informed consent if they meet the following inclusion criteria.

• Presenting with fever (≥37.5°C) and/or one mild symptom suggestive of COVID-19 disease (cough, dyspnoea, chills, odynophagia, diarrhoea, muscle pain, anosmia, dysgeusia, headache) OR being contact* of a SARS-CoV-2 confirmed or suspected case in the past 14 days

• More than 12 weeks of gestation (dated by ultrasonography)

• Agreement to deliver in the study hospitals

*Exclusion criteria*

• Known hypersensitivity to HCQ or other 4-amonoquinoline compounds

• History of retinopathy of any aetiology

• Concomitant use of digoxin, cyclosporine, cimetidine

• Known liver disease

• Clinical history of cardiac pathology including known long QT syndrome

• Unable to cooperate with the requirements of the study

• Participating in other intervention studies

• Delivery onset (characterized by painful uterine contractions and variable changes of the cervix, including some degree of effacement and slower progression of dilatation up to 5 cm for first and subsequent labours)

The study participants will be stratified by clinical presentation and SARS-CoV-2 PCR results. Assignment of participants to study groups will be as follows:

**• SARS-CoV-2-PCR confirmed, infected** pregnant women:

a. *symptomatic* (n=100)

b. *asymptomatic* (n=100)

**• SARS-CoV-2 PCR negative** pregnant women in contact* with a SARS-CoV-2-infected confirmed or suspected case (n=514).

*The ECDC definition of close contact will be followed.

The trial will be conducted in five hospitals in Spain: Hospital Clínic of Barcelona, Hospital Sant Joan de Déu and Hospital de la Santa Creu i Sant Pau, in Barcelona, and HM Puerta del Sur and Hospital Universitario de Torrejón, in Madrid.

**Intervention and comparator:**

Participants will be randomized to HCQ (400 mg/day for three days, followed by 200 mg/day for 11 days) or placebo (2 tablets for three days, followed by one tablet for 11 days).

**Main outcomes:**

The primary outcome is the number of PCR-confirmed infected pregnant women assessed from collected nasopharyngeal and oropharyngeal swabs at day 21 after treatment start (one week after treatment is completed).

**Randomisation:**

Allocation of participants to study arms will be done centrally by the trial’s Sponsor (the Barcelona Institute for Global Health, ISGlobal) by block randomization. This method will ensure balanced allocation to both arms. The electronic CRF will automatically assign a study number to each participant, depending on her study group and recruitment site. Each number will be related to a treatment number, which assigns them to one of the study arms.

**Blinding (masking):**

Participants, caregivers, investigators and those assessing the outcomes will be blinded to group assignment. Study tablets (HCQ and placebo) will be identically packaged in small opaque bottles.

**Numbers to be randomised (sample size):**

This study requires 200 SARS-CoV-2 infected and 514 contact pregnant women, randomised 1:1 with 100 and 227 respectively in each study arm.

**Trial Status:**

Protocol version 1.0, from May 8^th^, 2020. Recruitment is ongoing (first patient recruited the 19^th^ May 2020 and recruitment end anticipated by December 2020).

**Trial registration:**

EudraCT number: 2020-001587-29, registered 2 April 2020.

Clinicaltrials.gov identifier: NCT04410562, retrospectively registered 1 June 2020.

**Full protocol:**

The full protocol is attached as an additional file, accessible from the Trials website (Additional file 1). In the interest in expediting dissemination of this material, the familiar formatting has been eliminated; this Letter serves as a summary of the key elements of the full protocol.

## Supplementary information

**Additional file 1.** Full Study Protocol.

## Data Availability

All data will be available to the research collaborators. Decision to publish rests solely with the researchers. The full data set will be made publicly available not later than six months after trial completion.

