# Supplementary material for: Hydroxychloroquine efficacy and safety in preventing SARS-CoV-2 infection and COVID-19 disease severity during pregnancy (COVID-Preg): a structured summary of a study protocol for a randomised placebo controlled trial
Source: Trials. 2020 Jul 2;21:607. doi: 10.1186/s13063-020-04557-y (PMC7330539; doi:10.1186/s13063-020-04557-y)
Supplement: Supplementary file 1 — Additional file 1. Full Study Protocol. [file 13063_2020_4557_MOESM1_ESM.pdf]

# Hydroxychloroquine efficacy and safety in preventing SARS-CoV-2 infection and COVID-19 disease severity during pregnancy

**COVID-Preg (COVID**-19 during **Preg**nancy)

**Protocol v. 01**

**Dated: 8th May 2020**

## Summary of the Clinical Trial

| | |
|---|---|
| **EudraCT code** | 2020-001587-29 |
| **Funding** | **Instituto de Salud Carlos III.** Expresiones de interés para la financiación extraordinaria de proyectos de investigación sobre el SARS-COV-2 y la enfermedad COVID-19.<br>Project code: 286 |
| **Date** | 8th May 2020 |
| **Version** | Protocol version 01 |
| **Title** | Hydroxychloroquine efficacy and safety in preventing SARS-CoV-2 infection and COVID-19 disease severity during pregnancy |
| **Country** | Spain |
| **Study drugs** | Hydroxychloroquine: 400mg/day during three days, followed by 200mg/day during 11 days<br>Placebo: two tablets during three days, followed by 1 tablet/day during 11 days |
| **Intervention duration** | 14 days, followed by a follow-up visit 7 days post intervention (day 21) |
| **Study drugs provided by** | Laboratorios Rubió |
| **Phase of clinical trial** | Phase IIIb |
| **Main objectives** | 1. To assess the effect of HCQ in reducing SARS-CoV-2 viral shedding by PCR in infected pregnant women with mild symptoms.<br>2. To assess the efficacy of HCQ to prevent incident SARS-CoV-2 infection in pregnant women in contact with an infected or suspected case.<br>3. To evaluate the effect of HCQ in preventing the development of the COVID-19 disease in asymptomatic SARS-CoV-2-infected pregnant women. |
| **Study design** | Randomized double-blind placebo-controlled multicentre clinical trial |
| **Study population and sample size** | Pregnant women with and without mild symptoms/signs of SARS-CoV-2 infection and close contacts of confirmed or suspected cases and meeting inclusion criteria.<br>714 will be recruited |
| **Disease in study** | SARS-CoV-2 infection and COVID-19 disease |
| **Ethics committee that evaluated and approved the clinical trial** | Ethics Committee of the Hospital Clinic de Barcelona |
| **Sponsor** | **Barcelona Institute for Global Health (ISGlobal),**<br>Hospital Clínic - Universitat de Barcelona<br>Carrer Rosselló 132<br>E-08036 Barcelona, Spain |

## Study investigators, collaborators, institutions and other key positions

| | |
|---|---|
| **Principal Investigator** | Clara Menéndez, MD, PhD<br>Barcelona Institute for Global Health **(ISGlobal),**<br>Hospital Clínic- Universitat de Barcelona, Spain<br>Email: clara.menendez@isglobal.org |
| **ISGlobal Coordinating team** | Project manager: Clara Pons Duran, MSc<br>Email: clara.pons@isglobal.org<br><br>Project assistant: Haily Chen, MSc<br>Email: haily.chen@isglobal.org<br><br>Clinical coordinator: Laura Garcia-Otero, MD, PhD<br>Email: laura.garcia@isglobal.org<br><br>Technical coordinator: Raquel González, MD, MPH, PhD<br>Email: raquel.gonzalez@isglobal.org<br><br>Scientific advisor: Azucena Bardají, MD, PhD<br>Email: azucena.bardaji@isglobal.org<br><br>Communication manager: Elena Marbán Castro, MSc;<br>Email: elena.marban@isglobal.org<br><br>Data manager: Maximo Ramírez, MSc<br>Email: maximo.ramirez@isglobal.org<br><br>Safety coordinator: Luis Herrera, MD<br>Email: luisbernardo.herrera@isglobal.org |
| **Co-Principal Investigators** | Anna Goncé, MD, PhD<br>BCNatal \| Barcelona center for maternal fetal and neonatal medicine. Hospital Clínic and Hospital Sant Joan de Déu. Universitat de Barcelona.<br>Barcelona, Spain.<br>Email: agonce@clinic.cat<br><br>Elisa Llurba, MD, PhD<br>Obstetrics and Gynecology Department<br>Universitat Autònoma de Barcelona<br>Santa Creu i Sant Pau Hospital, Barcelona<br>Email: ellurba@santpau.cat<br><br>Maria del Mar Gil, MD, PhD<br>Obstetrics and Gynecology Department<br>Hospital Universitario de Torrejón, Madrid<br>Universidad Francisco de Vitoria, Madrid<br>Email: mariadelmar.gil@ufv.es<br><br>Miguel Ángel Rodríguez Zambrano, MD<br>HM Puerta del Sur, Móstoles, Madrid<br>Email: mrodzambrano@gmail.com |

| | |
|---|---|
| | M Dolores Gómez Roig, MD<br>BCNatal \| Barcelona Center for Maternal Fetal and Neonatal Medicine. Hospital Sant Joan de Déu and Hospital Clínic, University of Barcelona<br>Email: lgomezroig@sjdhospitalbarcelona.org |
| **Recruiting sites** | • BCNATAL \| Barcelona center for maternal fetal and neonatal medicine. Hospital Clínic and Hospital Sant Joan de Déu. Universitat de Barcelona. Barcelona, Spain<br>• Hospital Santa Creu i Sant Pau, Universitat Autònoma de Barcelona<br>• HM Puerta del Sur, Móstoles, Madrid<br>• Hospital Universitario de Torrejón, Universidad Francisco de Vitoria, Madrid |
| **Clinical monitor** | Designated person from the CRO **Dynamic Solutions S.L.** |
| **Data and Safety Monitoring Board Members** | Joan Albert Arnaiz (chair)<br>Clinical pharmacologist - Hospital Clínic of Barcelona;<br>Email: JAARNAIZ@clinic.cat<br><br>Montse Palacio, MD<br>Obstetrician, Hospital Clínic of Barcelona<br>Email: MPALACIO@clinic.cat<br><br>Ferran Torres<br>Statistitian; IDIBAPS<br>Email: ferran.torres@idibaps.org |
| **Methodological experts of the Sponsor** | Julia del Amo Valero, MD, PhD<br>Director of the National AIDS Plan<br>Dirección General de Salud Pública, Calidad e Innovación - Ministerio de Sanidad de España<br>Email: jamo@mscbs.es<br><br>Xavier Carné Cladellas, MD, PhD<br>Clinical pharmacologist<br>Email: xcarne.cladellas@gmail.com<br><br>Franco Pagnoni, MD<br>Scientific and cardiologist advisor<br>Barcelona Institute for Global Health (ISGlobal)<br>Email: franco.pagnoni@isglobal.org<br><br>Josep Brugada, MD, PhD<br>Cardiologist expert ; Hospital Clínic of Barcelona<br>Email : jbrugada@clinic.cat<br><br>Mª Ángeles Marcos, MD, PhD<br>Virologist; Hospital Clínic of Barcelona<br>Email: MMARCOS@clinic.cat |

## Table of Contents


List of abbreviations ........ 6
Synopsis ........ 7
1. Background ........ 9
2. Justification ........ 10
3. Hypotheses ........ 10
4. Objectives ........ 10
5. Methods ........ 11
   a. Study design ........ 11
      Study sites ........ 11
      Trial design ........ 11
      Primary endpoint ........ 11
      Secondary endpoints ........ 11
   b. Definitions ........ 13
   c. Study procedures ........ 14
   d. Laboratory procedures ........ 18
   e. Safety ........ 18
      HCQ side effects ........ 19
      Adverse Events ........ 19
      Severity, relationship of event to study drug, and outcome ........ 20
      Serious Adverse Events (SAE) ........ 21
      Data Safety Monitoring Board (DSMB) ........ 21
      Monitoring and reporting of Adverse Events ........ 22
   f. Data management ........ 22
   g. Quality assurance and quality control ........ 23
   h. Trial monitoring ........ 23
   **i.** Data analysis ........ 24
      Statistical analysis populations ........ 24
      Patient demographic data and other baseline characteristics ........ 24
6. Benefit/risk assessment ........ 25
7. Ethical considerations ........ 25
   a. Human participants involved ........ 25
   b. Informed consent ........ 25
   c. Inclusion of vulnerable populations ........ 26
   d. Collection of biological samples ........ 26
   e. Data protection ........ 26
   f. Ethical and regulatory approval ........ 26
8. Administrative aspects ........ 27
9. Budget ........ 27
10. Dissemination of results ........ 27
11. Timeline ........ 27
12. References ........ 28

## List of abbreviations

| | |
|---|---|
| ANC | Antenatal care |
| ECG | Electrocardiogram |
| HCQ | Hydroxychloroquine |
| US | Ultrasound |
| WHO | World Health Organization |
| eCRF | Electronic Case Report Form |
| PCR | Polymerase Chain Reaction |

## Synopsis

The emergence of severe acute respiratory syndrome coronavirus 2 (SARS-CoV-2) disease (COVID-19) in China at the end of 2019 has caused a large global outbreak and is a major world public health issue. Since the onset of the SARS-CoV-2 epidemic, very few studies have reported on the effects of the infection on maternal health. Importantly, pregnant women should be considered a population at increased risk of infection, given the greater risk of severe morbidity and mortality from SARS and influenza. Pregnancy is considered a period of susceptibility for infections, including respiratory pathogens and severe pneumonia. Regarding SARS-CoV-2 it is still unclear how the infection affects pregnant women and their offspring, as well as which factors may influence maternal disease and outcomes.

This is a randomized, double blinded, placebo-controlled multicentre clinical trial including 714 pregnant women (200 SARS-CoV-2 infected -100 symptomatic with mild disease and 100 asymptomatic- pregnant women and 514 SARS-CoV-2 uninfected pregnant women who are contacts with a SARS-CoV-2 case) with the main objectives of assessing the safety and efficacy of oral hydroxychloroquine (HCQ) in reducing maternal viral shedding by PCR, and preventing incident SARS-CoV-2 infection and disease severity. Pregnant women undergoing antenatal follow up at five maternity hospitals, presenting at least one sign and/or one mild suggestive symptoms and a positive SARS-CoV-2 PCR test, or who are contacts of a suspected or confirmed case, will be recruited and randomised 1:1 to receive HCQ orally (400 mg/day for 3 days, followed by 200 mg/day for 11 days) or placebo. Women will be followed up for the duration of the intervention. One week after intervention completion, a SARS-CoV-2 PCR test will be repeated. At delivery, the pregnancy outcome will be registered and a cord blood sample will be collected to measure for IgG and IgM of SARS-CoV-2. A neonatal nasopharyngeal aspirate will be collected to perform PCR SARS-CoV-2 testing.

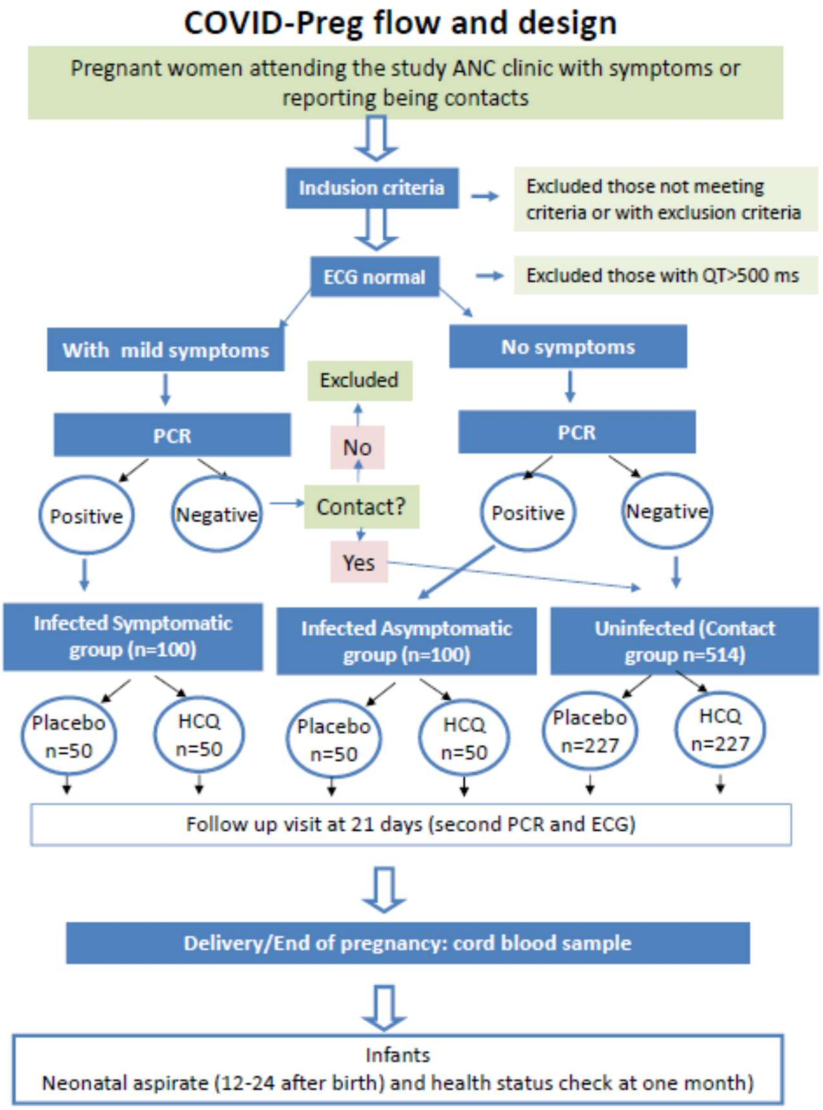
COVID-Preg flow and design
Pregnant women attending the study ANC clinic with symptoms or reporting being contacts
Inclusion criteria
Excluded those not meeting criteria or with exclusion criteria
ECG normal
Excluded those with QT>500 ms
With mild symptoms
No symptoms
Excluded
PCR
No
PCR
Positive
Negative
Contact?
Positive
Negative
Yes
Infected Symptomatic group (n=100)
Infected Asymptomatic group (n=100)
Uninfected (Contact group n=514)
Placebo n=50
HCQ n=50
Placebo n=50
HCQ n=50
Placebo n=227
HCQ n=227
Follow up visit at 21 days (second PCR and ECG)
Delivery/End of pregnancy: cord blood sample
Infants
Neonatal aspirate (12-24 after birth) and health status check at one month)

## 1. Background

The outbreak of severe acute respiratory syndrome coronavirus 2 (SARS-CoV-2) and its disease named COVID-19 that started in November 2019 in Wuhan (China) has rapidly spread worldwide (1) and was declared a pandemic of Public Health Emergency of International Concern by the World Health Organization (WHO) in March 2020.

Globally, epidemiological data is being reported as total number of laboratory-confirmed cases and associated deaths; but limited information exists regarding the prevalence and incidence of infection among pregnant women. Additionally, there is insufficient data to estimate if pregnant women are at an increased risk of getting infected from, and/or to develop severe health outcomes, compared to the general population (2).

Pregnancy is a period of susceptibility for infections, including respiratory pathogens and severe pneumonia (3,4). Thus, pneumonia from any infectious aetiology is an important cause of morbidity and mortality in pregnant women, being reported as the 2$^{nd}$ and 3$^{rd}$ most common cause of indirect maternal death in some studies (5,6). It is estimated that about 25% of pregnant women that develop pneumonia will need to be hospitalized in critical care units and require ventilatory support (7). Although bacterial pneumonia may be a serious disease when it occurs during pregnancy viral pneumonia has even higher levels of morbidity and mortality during pregnancy (8). Differential severity and mortality rates in pregnant patients have been reported in epidemics, such as the 1918 influenza pandemic (27% in pregnancy vs. 2,6% in non-pregnant adults-higher if infected in the 3$^{rd}$ trimester-) (9), 2009 H1N1 influenza (4 times more likely to be admitted to hospital) (10), and 2003 SARS (50% of pregnant women infected were admitted to intensive care, 33% required mechanical ventilation, and 25% mortality rate) (11). The commonest adverse obstetrical outcomes associated with maternal pneumonia from all causes include preterm labour, intrauterine growth restriction (IUGR), stillbirth and neonatal death (7).

The limited information regarding the effects of coronavirus infections during pregnancy results from the epidemics of severe acute respiratory syndrome (SARS) and the Middle East respiratory syndrome (MERS) (12). These two diseases might provide insights into the effects in pregnancy of the new epidemic of coronavirus disease 2019 (COVID-19). The experience with SARS and MERS suggests that both infections are associated with poor maternal and fetal-neonatal outcomes, including severe maternal disease requiring hospitalization, intensive care and ventilator support as well as preterm delivery, IUGR, spontaneous abortion and perinatal death; importantly, the data available indicates that the severity of the disease is worse in pregnant women than in infected non-pregnant women (3). On the other hand, intrauterine maternal-fetal transmission of these two infections has not been documented (3).

Regarding COVID-19, very limited data still exists about its effects in pregnancy. The few reports available of pregnant women infected with COVID-19, all were infected in the third trimester and clinical features were similar to those in non-pregnant adults, with fetal distress and preterm delivery seen in some cases. All women delivered by caesarean section and the newborns tested negative for SARS-CoV-2 (3).

On the other hand, according to the World Health Organisation, there is currently no medication shown to be effective for the treatment or prevention of the SARS-CoV-2 (13).

Early *in-vitro* studies after the SARS 2003 epidemic showed that chloroquine (CQ) had a strong in-vitro antiviral effect on SARS-CoV infection of primate cells (14)  (13). More recently, an inhibitory *in-vitro* effect of CQ has been shown on the SARS-CoV-2 (15). In addition, *in-vitro* studies comparing CQ with hydroxychloroquine (HCQ) on SARS-CoV-2 infected cells showed that HCQ had a more potent inhibitory effect than CQ (14). These findings led to clinical studies in

China with encouraging results and to include CQ in the recommendations regarding prevention and treatment of the infection in this country (16). In other countries suffering the epidemic (such as Spain) HCQ is currently used to treat admitted patients with SARS-CoV-2 infection. Preliminary unpublished results of a French clinical trial in 24 infected patients showed a clearance of the virus in 75% of those treated with HCQ for 6 days compared with 10% in untreated controls (17)

Chloroquine and HCQ are used in the treatment of systemic lupus erythematosus (SLE) and rheumatoid arthritis (RA) for more than 25 years including in pregnant women (18). The safety of this treatment has been well established during pregnancy. In addition, there is wide evidence of the safety of CQ in the treatment and prevention of malaria in pregnancy in endemic areas (19). Complete rationale for drug selection and dosage are included in Annex 1.

## 2. Justification

It still unclear how SARS-CoV-2 affects pregnant women and their offspring, as well as which factors may influence obstetrical disease and outcomes, including the timing of maternal viral exposure by gestational age, the effects of parity, age, host immune responses, coexisting medical and obstetrical conditions and the effects of treatment regimens. While further information is gathered, based on the existing evidence from other infections causing pneumonia, pregnant women should be considered to be at high risk for developing severe infection during the current COVID-19 epidemic. Results from clinical trials with HCQ in non-pregnant adults may not be directly extrapolated to pregnant women given the special features of the pregnancy status.

Thus, clinical research is urgently needed to improve the care and reduce the risk of poor pregnancy outcomes of women in this and in future epidemics.

## 3. Hypotheses

- In SARS-CoV-2-infected pregnant women with non-severe COVID-19 disease, the administration of oral hydroxychloroquine for 14 days reduces viral shedding by PCR and disease progression.
- In SARS-CoV-2-infected asymptomatic pregnant women, the administration of oral hydroxychloroquine for 14 days prevents the development of COVID-19 disease.
- In SARS-CoV-2 PCR negative pregnant women in contact with a SARS-CoV-2-infected or suspected case, the administration of oral hydroxychloroquine for 14 days prevents SARS-CoV-2 infection.

## 4. Objectives

**Primary**

1. To assess the effect of HCQ in reducing SARS-CoV-2 viral shedding by PCR in infected pregnant women with mild symptoms.
2. To assess the efficacy of HCQ to prevent incident SARS-CoV-2 infection in pregnant women in contact with an infected or suspected case.
3. To evaluate the effect of HCQ in preventing the development of the COVID-19 disease in asymptomatic SARS-CoV-2-infected pregnant women.

**Secondary**

1. To determine the effect of HCQ on the clinical course and duration of the COVID-19 disease in SARS-CoV-2-infected pregnant women.
2. To determine the impact of HCQ on the risk of hospitalization and mortality of SARS-CoV-2-infected pregnant women.
3. To assess the safety and tolerability of HCQ in pregnant women.

4. To describe the clinical presentation of SARS-CoV-2 infection during pregnancy.
5. To describe the effects of maternal SARS-CoV-2 infection on pregnancy and perinatal outcomes by treatment group.
6. To determine the risk of vertical transmission (intra-utero and intra-partum) of SARS-CoV-2.

## 5. Methods

### a. Study design

This is a randomized controlled clinical trial.

#### Study sites

The trial will be conducted in five hospitals in Spain: the Hospital Clínic of Barcelona, the Hospital Sant Joan de Déu and the Hospital de la Santa Creu i Sant Pau, in Barcelona, and the HM Puerta del Sur and Hospital Universitario de Torrejón, in Madrid. Madrid and Catalonia are the two regions more severely affected by the COVID-19 pandemic in Spain, with 56963 and 41676 reported cases respectively at date 20th April 2020 (20).

#### Trial design

The trial has been designed as a randomized double-blind placebo-controlled multicentre trial to evaluate the safety and efficacy of HCQ to prevent and/or minimize SARS-CoV-2 infection during pregnancy. A total of 714 pregnant women are expected to participate in the study (see sample size calculation).

The study participants will be stratified by clinical presentation and SARS-CoV-2 PCR results. The following are the study groups:

- **SARS-CoV-2-PCR confirmed, infected** pregnant women:
    a. *symptomatic* (n=100)
    b. *asymptomatic* (n=100)
- **SARS-CoV-2 PCR negative** pregnant women in **contact** with a SARS-CoV-2-infected confirmed or suspected case (n=514).

Women in each group will be randomly allocated to receive either HCQ or placebo. Evaluations will include safety and tolerability, SARS-CoV-2 PCR and COVID-19 clinical diagnosis and presentation, as well as neonatal SARS-CoV-2 by PCR and IgM/IgG.

#### Primary endpoint

Number of PCR-confirmed infected pregnant women assessed from collected nasopharyngeal and oropharyngeal swabs at day 21 after treatment start (one week after treatment is completed).

#### Secondary endpoints

*Maternal/Foetal*

1. Incidence of COVID-19 disease during pregnancy
2. Incidence of COVID-19-related admissions
3. Incidence of all-cause admissions
4. Incidence of all-cause outpatient attendances
5. Mean duration of symptoms-signs of COVID-19

6. Frequency and severity of adverse events
7. Incidence of preeclampsia
8. Incidence of gestational diabetes
9. Incidence of SARS-CoV-2 infections during pregnancy
10. Prevalence of intrauterine growth restriction (<10$^{th}$ centile according to local standards) (21)
11. Maternal mortality rate

*Infant*

1. Proportion of neonates with SARS-CoV-2- intrauterine infection by PCR-confirmed SARS-CoV-2-infection in nasopharyngeal aspirate.
2. Proportion of neonates with clinical signs/symptoms of COVID-19
3. Prevalence of low birth weight (<10$^{th}$ centile according to local standards) (21)
4. Prevalence of preterm birth (<37 weeks of gestational age)
5. Prevalence of embryo and foetal losses (miscarriages and stillbirths)
6. Frequency of congenital malformations
7. Proportion of adverse perinatal outcome
8. Neonatal morbidity
9. Neonatal mortality rate

## Sample size

A sample size of 100 infected pregnant women of each asymptomatic and symptomatic group, 1:1 randomly allocated into two study arms, allows to detect an effect size of 30%, 34% and 39% with 80% power and two-tail type-I error of 0.05 assuming 3%, 10% and 30% curation rates in the control group, respectively. In other words, this sample allows detecting differences if the cure rates in the intervention group are at least 33%, 44%, and 69%, respectively. A sample size of 257 non-infected, contact positive, pregnant women per intervention arm will be needed to detect a 3 % reduction in the prevalence of infection with 80% power and two-tail type-I error of 0.05, assuming a prevalence of infection in the control group of 3% after two weeks of follow up.

## Study population

Pregnant women who are undergoing routinely prenatal follow up or attend emergency units at the recruitment hospitals and who report symptoms/signs suggestive of COVID-19 disease or being contact of a suspected or confirmed COVID-19 case.

*Inclusion criteria*

Women will be invited to participate in the trial and sign an informed consent if they meet the following inclusion criteria.

- Presenting with fever (≥37.5ºC) and/or one mild symptom suggestive of COVID-19 disease (cough, dyspnoea, chills, odynophagia, diarrhoea, muscle pain, anosmia, taste disorder, headache) OR contact of a SARS-CoV-2 confirmed or suspected case in the past 14 days
- More than 12 weeks of gestation (dated by ultrasonography)
- Agreement to deliver in the study hospitals

*Exclusion criteria*

- Known hypersensitivity to HCQ or other 4-amonoquinoline compounds
- History of retinopathy of any aetiology
- Concomitant use of digoxin, cyclosporine, cimetidine

- Known liver disease
- Clinical history of cardiac pathology including known long QT syndrome
- Unable to cooperate with the requirements of the study
- Participating in other intervention studies
- Delivery onset (characterized by painful uterine contractions and variable changes of the cervix, including some degree of effacement and slower progression of dilatation up to 5 cm for first and subsequent labours)

### b. Definitions

***Contact***

According to the WHO and the European Centre for Disease Prevention and Control (EDCD) a contact of a COVID-19 case is any person who has had contact with a COVID-19 case (Annex 2) within a timeframe ranging from 48 hours before the onset of symptoms of the case to 14 days after the onset of symptoms (22).

If the case had no symptoms, a contact person is defined as someone who has had contact with the case within a timeframe ranging from 48 hours before the sample which led to confirmation was taken, to 14 days after the sample was taken. The associated risk of infection depends on the level of exposure.

Since local and community transmission has been reported in Spain and in the study areas, all patients presenting with symptoms of acute respiratory infection in primary care or the emergency department of a hospital (first contact with the healthcare system) will be considered as suspected cases.

***Probable case***

A suspected case for whom testing for virus causing COVID-19 is inconclusive (according to the test results reported by the laboratory) or for whom testing was positive on a pan-coronavirus assay.

***Confirmed case***

A person with laboratory confirmation of virus causing COVID-19 infection, irrespective of clinical signs and symptoms.

***Clinical severity***

The definitions for mild and severe illness are in Annex 3, while those with critical illness are defined as patients with acute respiratory distress syndrome or sepsis with acute organ dysfunction. Mild illness will be considered in case of patients with uncomplicated upper respiratory tract viral infection, or non-specific symptoms such as fever, fatigue, cough (with or without sputum production), malaise, muscle pain, sore throat or dyspnea. Patients may also present with diarrhoea, headache, anosmia or taste disorders or non severe pneumonia (suspected respiratory infection without severity criteria -respiratory rate > 30 breaths/min; severe respiratory distress; or $SpO2 \leq 93\%$ on room air).

***Prolonged QTc interval***

The QT interval is dependent on the heart rate and should be adjusted to improve the detection of patients at increased risk of ventricular arrhythmia. The most commonly used QT correction formula is the Bazett's formula, calculating the heart rate-corrected QT interval (QTcB). Bazett's formula is often given in a form that returns QTc in dimensionally suspect units, square root of

seconds. The mathematically correct form of Bazett's formula is: QTcB=QT/ √RR. A QTc above 500 ms and/or QTc ≥25% from baseline. will be considered abnormal (23).

### c. Study procedures

#### Enrolment

Participants will be enrolled in the study following the steps described below.

***a) Screening***

At each hospital several recruitment approaches will be used, including:

- Prenatal antenatal care and routine prenatal ultrasonography
- The emergency room at the study hospitals
- Referrals from other providers SARS-CoV-2 testing sites and laboratories
- Use of online and social networking websites and apps

Pregnant women who undergo routine follow up at the recruitment hospitals (prenatal visits or obstetric screening ultrasounds) will be provided an informative study leaflet and be invited for study eligibility assessment while they are waiting to be attended. Study personnel will explain the study details to the women who accept to participate in the screening procedures.

***b) Informed Consent***

Those women who fulfil the inclusion criteria and accept to participate will be asked to sign an informed consent form. This will be obtained before any study procedures and tests are initiated. The informed consent will cover the woman and the newborn (Annex 4).

***c) Electrocardiagram***

An electrocardiogram (ECG) will be performed using a mobile device (Kardia©). If the QTc is > than 500 milliseconds the woman with be considered as “study screening failure” and she will not be included in the study. If the QTc is < than 500 ms the recruitment procedures will continue.

***d) Demographic, obstetric and medical data***

The woman’s demographic and medical and obstetric data will be recorded onto a specific electronic case report form (eCRF). The physical examination of the woman at the ANC visit will be recorded and will include the following assessments: weight, height, gestational age by first trimester ultrasound assessment.

***e) SARS-CoV-2 testing***

A nasopharyngeal and oropharyngeal swabs of all included women will be collected for SARS-CoV-2 diagnosis by PCR. Additionally, 5 ml of venous blood will be collected for SARS-CoV-2 serologic analysis of all participants at baseline.

#### Randomization

***a) Study groups***

Based on the presence of signs and symptoms and SARS-CoV-2 PCR results, participants will be assigned to one of the following study groups:

- Symptomatic women (mild disease)
    - PCR-confirmed SARS-CoV-2 infected → will be considered symptomatic infected

- PCR-negative SARS-CoV-2 -> will be considered not infected and will be withdrawn from the study unless reporting having been in contact with a COVID-19 confirmed or suspected case in the previous 14 days

- Asymptomatic women who are contacts with a confirmed or suspected case
  - PCR-confirmed SARS-CoV-2 infected → will be considered asymptomatic infected
  - PCR-negative SARS-CoV-2 infected → will be considered contact uninfected

***b) Treatment arm allocation***

Participants will be then randomized in a 1:1 ratio to HCQ (400 mg/day for three days, followed by 200 mg/day for 11 days) or placebo (2 tablets for three days, followed by one tablet for 11 days).

Allocation of participants to study arms will be done centrally by the trial's Sponsor (the Barcelona Institute for Global Health, ISGlobal) by block randomization. This method will ensure balanced allocation to both arms. The electronic CRF will assign automatically a study number to each participant, depending on her study group and recruitment site. Each number will be related to a treatment number, which assigns them to one of the study arms. Randomizations lists with treatment assignment for each study participant will be concealed in opaque sealed envelopes and only open in case of urgent need following study unblinding procedures.

A study card will be given to the participant in order to facilitate their clinical management in case they attend primary care centres or other hospitals.

### Administration of study drugs

Study tablets will be identically packaged in small opaque bottles. All study personnel, investigators and the participants will remain blinded throughout the trial.

Participants will receive a bottle containing 19 tablets of study medication. They will be instructed to take two tablets for first three days and one tablet for the following 11 days. The first day of treatment, drug administration will be supervised.

Women vomiting within the first 30 minutes of drug administration will be given a second full drug dose; women vomiting after 30-60 minutes of study tablets administration will be given an additional half dose of the drug.

### Follow-up and assessment of treatment adherence

Participants will be contacted by phone daily during the intervention period by study personnel to assess general health condition, as well as the drugs' tolerability and potential adverse events. Treatment adherence and the intake of any concomitant medication will be also requested daily. Information regarding any unscheduled study visit to the hospital will be registered.

### Household and follow-up visits

At day 21 after initiating study drugs' administration (one week after treatment completion), study personnel will visit women at home or at the hospital to collect a nasopharyngeal and oropharyngeal swabs for PCR -SARS-CoV-2 and to perform an ECG.

The study personnel will assess treatment adherence by requesting and counting if there are remaining study pills.

### End of pregnancy/ New-born assessments

***a) Maternal clinical examination and assessments***

At the time of end of pregnancy (delivery or miscarriage) the outcome of the pregnancy will be recorded onto the study questionnaires.

***b) Placental sample collection***

In a sub-sample of study participants (n=120, randomly selected from the three study groups), a placental sample will be collected for histological examination. Within minutes of placental expulsion, the fetal surface of the placenta will be disinfected and incised with a sterile scalpel, and 2 swabs and biopsies (close to the umbilical cord and peripheral margin) will be obtained.

***c) New-born assessments***

A 5 ml blood sample for SARS-CoV-2 for Ig G and Ig M determination will be collected from the umbilical vein once the baby's umbilical cord has been cut and clamped. Information on the new-born anthropometrical parameters and clinical status will be recorded onto the study eCRF.

A nasopharyngeal aspirate will be collected 12 to 24 hours after birth for PCR- SARS-CoV-2 infection assessment.

***d) Miscarriage and other adverse pregnancy outcomes***

If the outcome of the pregnancy is a miscarriage or stillbirth, women will be visited at the study facilities and the same study procedures will be applied.

### Infant's check at one month of age

The health status on the infant will be checked by a telematics clinical visit at one month of age.

### Unscheduled visits

Study participants reporting sick at the phone or home visits will be recommended to contact the study hospitals by phone and follow their instructions. Every unscheduled visit of the woman from enrolment until delivery will be recorded onto the study eCRF.

***a) Physical and clinical examination***

Gestational age, blood pressure and axillary temperature will be registered. Suggestive symptoms of SARS-CoV-2 infection will be recorded

***b) Other medications***

Any concomitant treatment or history of treatment since the last visit will be recorded in the study eCRF. In addition, information on adverse events related to study drugs will be collected.

### Table 1. Study visits and procedures

| Study procedure | Enrolment/ randomisation | Follow-up visits during intervention | Household visit (7 days after treatment' completion) | Pregnancy follow-up | Delivery/ End of pregnancy | One month after birth |
|---|---|---|---|---|---|---|
| Inclusion/exclusion criteria check | x | | | | | |
| Written informed consent | x | | | | | |
| ECG | x | | x | | | |
| Demographics/medical history | x | | | x | | |
| Physical/clinical examination | x | | | x | | |
| Concomitant medication | x | | x | x | x | |
| Gestational age | x | | x | x | x | |
| • Nasopharyngeal and oropharyngeal swabs for maternal SARS-CoV-2 PCR<br>• Venous blood (5 ml, IgG and IgM against SARS-CoV-2) | x | | x | | | |
| Maternal and perinatal outcome | | | | | x | |
| Umbilical cord blood (IgG and IgM against SARS-CoV-2) | | | | | x | |
| Placenta sample (histological examination) | | | | | X** | |
| Neonatal nasopharyngeal aspirate*** | | | | | x | |
| Adverse events/Severe adverse events | | x | x | x | | |
| Tolerability | | x | x | | | |
| Signs and symptoms of COVID-19 | x | x | x | x | | |
| Adherence to study medication | | x | x | | | |
| Ultrasound data | | | | x | | |
| Infant health status check (telematics visit) | | | | | | X |

* In case of presence of fever and/or two symptoms suggestive of COVID-19 disease (fever, fatigue, cough (with or without sputum production), anorexia, malaise, muscle pain, sore throat, dyspnea, nasal congestion, or headache,diarrhoea, nausea and vomiting) in the previous 14 days, women will be collected a nasopharyngeal and oropharyngeal swabs for PCR analysis at any time.

**In a sub-sample of participants (n=120).

***12-24 hours of life.

### d. Laboratory procedures

#### Detection of SARS-CoV-2 infection

A Real-Time polymerase chain reaction (PCR) Coronavirus (COVID-19) assay diagnostic test will be performed at the lab study hospital for specific detection of SARS-CoV-2 viral RNA. Real-Time PCR technology utilizes polymerase chain reaction for the amplification of specific target sequences and target specific probes for the detection of the amplified RNA. The probes are labelled with fluorescent reporter and quencher dyes.

*Principles of RT-PCR SARS-CoV-2 procedures*

The oligonucleotide primers and probes for detection of SARS-CoV-2 are selected from regions of the virus nucleocapsid (N) gene. The panel is designed for specific detection of the SARS-CoV-2 (two primer/probe sets). An additional primer/probe set to detect the human RNase P gene (RP) in control samples and clinical specimens is also included in the panel. RNA isolated and purified from upper and lower respiratory specimens is reverse transcribed to cDNA and subsequently amplified in the Applied Biosystems 7500 Fast Dx Real-Time PCR Instrument with SDS version 1.4 software. In the process, the probe anneals to a specific target sequence located between the forward and reverse primers. During the extension phase of the PCR cycle, the 5' nuclease activity of Taq polymerase degrades the probe, causing the reporter dye to separate from the quencher dye, generating a fluorescent signal. With each cycle, additional reporter dye molecules are cleaved from their respective probes, increasing the fluorescence intensity. Fluorescence intensity is monitored at each PCR cycle by Applied Biosystems 7500 Fast Dx Real-Time PCR System with SDS version 1.4 software (24–26).

*SARS-CoV-2 serological assays*

A multiplex high-performance immunoassay is being developed to quantify IgG and IgM antibodies that target fragments of the main SARS-CoV-2 protein interacting with the host, "spike" (S), through the Luminex technology already set in ISGlobal's laboratory. Luminex platform allows analysing a wide range of immunological responses in front of other viral antigens (membrane (M), envelope (E) and nucleocapside (N)) to increase the probabilities of serologically capturing the exposition and impact of the antigen diversity of the virus and its serological reactivity. Enzyme-linked immunosorbent assay (ELISA) or immunofluorescence assay (IFA) may also be used to detect IgG and IgM against SARS-CoV-2 in serum specimens of participants.

*Histological exam of the placenta*

A sub-sample of participants (n=120, randomly selected from the three study groups) will have a placental sample collected for histological analysis. The biopsies will be immediately placed in 25 mL of 10% neutral buffered formalin and kept at 4ºC until processed and embedded in paraffin wax by standard techniques. Paraffin sections will be stained with hematoxylin and eosin, Giemsa's stain and the periodic acid-Schiff technique. Placental histology will include the examination of inflammatory signs (such as presence of neutrophils and monocytes) in the subchorial space and the umbilical cord connective tissue (funisitis) and analysis of intervillous fibrin deposition (27).

### e. Safety

The study is designed as a clinical trial in which pregnant women followed up prospectively from the antenatal care clinic contact until the end of pregnancy and their newborns for the first 24 hours of life.

Drug exposure to any drug during pregnancy may carry risks to both the mother and the developing foetus (28). Chloroquine and HCQ are on the WHO Essential Medicines List for use in rheumatic disorders, and is widely prescribed as an anti-inflammatory for rheumatoid arthritis (RA), systemic lupus erythematosus (SLE), and other autoimmune syndromes for more than 25 years including in pregnant women (18). Therefore, the safety of this treatment has been well established during pregnancy. In addition, there is wide evidence of the safety of CQ in the treatment and prevention of malaria in pregnancy in endemic areas (19). HCQ is associated with a better safety profile for daily and chronic use than CQ. Based on the limited *in vitro* data available, HCQ appears to be slightly more potent than CQ against SARS-CoV-2 (15).

With tens of millions of doses administrated for malaria and autoimmune diseases, the side effect profile of HCQ is well described and the drug is generally well tolerated.

### HCQ side effects

With short-term administration (as opposed to chronic/years long use in rheumatologic disease management), the major AEs are gastrointestinal (nausea, vomiting, dyspepsia, abdominal cramps, and diarrhoea) and transient skin rashes. The gastrointestinal symptoms may vary by the specific generic manufacturer of HCQ, and are best managed by taking the drug with food or a glass of milk. A transient rash, most commonly morbilliform or psoriasiform, can develop in 10% of participants often with a sustained loading dose, and is often managed by lowering the dose. To avoid this potential side effect, this protocol is using a short loading dose, not a sustained one. Uncommonly, idiosyncratic leukopenia/ thrombocytopenia can occur and the drug should not be given to those with underlying bone marrow disorders. Lastly, hypoglycaemia can occur, and those taking insulin or glucose-lower drugs are at risk, and blood glucose should be monitored.

Manifestations of long term administration of HCQ, including retinitis, renal and hepatic disease, and cardiomyopathy are unlikely to occur in a short treatment duration such as the proposed 2-week exposure.

### Adverse Events

*Definition of an Adverse Event (AE):*

Any untoward medical occurrence in a subject to whom a medicinal product has been administered, including occurrences which are not necessarily caused by or related to that product.

An AE can therefore be any unfavourable and unintended sign (including a clinically significant abnormal finding), symptom or disease temporally associated with the use of a medicinal product, whether or not considered related to the medicinal product.

All AEs should be treated appropriately. Treatment may include one or more of the following:

- no action taken (i.e. further observation only)
- study drug dosage adjusted/temporarily interrupted
- study drug permanently discontinued due to this adverse event
- concomitant medication given
- non-drug therapy given
- patient hospitalized/patient's hospitalization prolonged.

The action taken to treat the adverse event will be recorded on the AE form. Once an adverse event is detected, it will be followed until its resolution or until it is judged to be permanent, and assessment will be made at each visit (or more frequently, if necessary) of any changes in

severity, the suspected relationship to the study drug, the interventions required to treat it, and the outcome.

Information about common side effects already known about the study drug will be included in the patient informed consent and will be discussed with the patient during the study as needed.

### Severity, relationship of event to study drug, and outcome

The **severity** of a clinical adverse event is to be scored according to the following scale:

| Grade | Severity | Description |
|---|---|---|
| **1** | Mild | **Awareness of sign or symptom, but easily tolerated** |
| **2** | Moderate | **Discomfort enough to cause interference with usual activity** |
| **3** | Severe | **Incapacitating with inability to work or perform usual activity** |
| **4** | **Life-threatening** | **Patients at risk of death at the time of the event** |

All adverse events will be assessed and documented by the local principal investigator using a toxicity table that will be developed as SOP. All grade 3-4 toxicities are considered **serious**.

Changes in the severity of an AE should be documented to allow an assessment of the duration of the event at each level of severity. Adverse events characterized as intermittent will provide documentation of onset and duration of each episode.

The **relationship** of an adverse event to study drug is to be assessed according to the following definitions:

*Definitely unrelated*:
It should be reserved for those events which occur prior to test drug administration (e.g., washout or single-blind placebo) or for those events which cannot be even remotely related to study participation (e.g. injury caused by a third party).

*Unlikely:*
There is no reasonable temporal association between the study drug and the suspected event and the event could have been produced by the subject's clinical state or other modes of therapy administered to the subject.

*Possible:*
The suspected adverse event may or may not follow a reasonable temporal sequence from study drug administration but seems to be the type of reaction that cannot be dismissed as unlikely. The event could have been produced or mimicked by the subject's clinical state or by other modes of therapy concomitantly administered to the subject.

*Probable*:
The suspected adverse event follows a reasonable temporal sequence from study drug administration, abates upon discontinuation of the drug, and cannot be reasonably explained by the known characteristics of the subject's clinical state.

*Definitely related*
It should be reserved for those events, which have no uncertainty in their relationship to test drug administration: this means that a re-challenge was positive.

The **outcome** of each AE must be assessed according to the following classification:

- Completely recovered: the patient has fully recovered with no observable residual effects
- Not yet completely recovered: improvement in the patient's condition has occurred, but the patient still has some residual effects
- Deterioration: the patient's overall condition has worsened
- Permanent damage: the AE has resulted in a permanent impairment
- Death: the patient died due to the AE
- Ongoing: the AE has not resolved and remains the same as at onset
- Unknown: The outcome of the AE is not known because the patient did not return for follow-up (lost to follow-up)

### Serious Adverse Events (SAE)

A SAE is an adverse event that:

- Results in death
- Is life threatening
- Requires hospitalization or prolongation of existing hospitalization
- Results in persistent or significant disability or incapacity or
- Consists of a congenital anomaly or birth defect

The Principal Investigators at each study site will be responsible for recording, management, and reporting of the AEs and SAEs. They must be recorded in the AE/SAE CRF with the following information:

- The severity grade (mild, moderate, severe)
- Relationship to the study drug(s) (suspected/not suspected)
- Duration (start and end dates or if continuing at final exam)

*Definition of Suspected Unexpected Serious Adverse Reaction (SUSAR):*
A serious adverse event (SAE) becomes a SUSAR (suspected unexpected serious adverse reaction) if the event is suspected (possibly, probably or definitely) to be related to the study drug and unexpected i.e. not previously documented in any of the product information or protocol.

### Data Safety Monitoring Board (DSMB)

A Data and Safety Monitoring Board (DSMB) will be constituted previously to trial's initiation to review and analyse all collected clinical safety data and to assess unexpected AEs and SAEs. The DSMB will be an independent committee consisting of experts in infectious diseases, obstetrics, epidemiology, pharmacology, biostatistics and other appropriate disciplines that will be appointed to oversee ethical and safety aspects of the conduct of the trial.

The DSMB will review the implementation and progress of the study, providing advice on safety-related issues before the initiation of the study, during the study and at the close out of the study. Its advice will be based on the interpretation of study data with reference to the study protocol. The DSMB will be empowered to put the study on hold pending review of potential safety issues.

The DSMB will be informed by the Safety Coordinator of all protocol amendments, informed consent changes or revisions of other documents originally submitted for review, of all subsequent protocol modifications (for information) and of new information that may adversely affect the safety of the subjects or the conduct of the study. The DSMB will also review and approve Safety Reports submitted by the Safety Coordinator. All documentation provided to members of the DSMB for information and review will be treated in a confidential manner.

### Monitoring and reporting of Adverse Events

Safety will be evaluated in the mother, the foetus and the newborn. Active safety monitoring will consist in daily phone visits to study participants from the initiation of treatment administration to assess drug tolerability and record all Adverse Events (AEs). In addition, a health facility-based passive surveillance system will be established to capture unscheduled visits of participants during follow-up.

The Principal Investigators (PI) at each study site will be responsible for recording, managing, and reporting of the AEs and SAEs as per study protocol. All AEs will be recorded in the AE form, and the DSMB will agree which SAEs and in what time frame will be reported.

All SAEs will be recorded on the SAE form and will be reported within 24 or 48 hours to the sponsor's (ISGlobal) Safety Coordinator, the CRO Dynamic Solutions, and to the DSMB as per study protocol. A flowchart will be provided to each study site to train the procedures for assessing AEs and reporting requirement according to severity and expectedness as well as persons responsible for recording and reporting of AEs/SAEs.

All participants enrolled in the trial will be educated at the beginning of the study on how to and whom to contact if they experience any untoward event and/or get admitted to the hospital.

In addition, Interim Safety Reports and Annual Safety Reports will be prepared by the Safety Coordinator and the CRO Dynamic Solutions and distributed to the local Ethic Committee, Sponsor, and DSMB as agreed in the study protocol.

The CRO Dynamic Solutions will ensure that AEs and SAEs are reported in compliance with International Conference on Harmonisation (ICH) Good Clinical Practice (GCP) guidelines and current international legislation during COVID-19 pandemic.

### f. Data management

Data will be directly collected and entered in the electronic database through mobile devices. Therefore, electronic Case Report Form (eCRFs) will be developed for capturing of the trial's data. As the CRO Dynamic Solutions will be in charge of the management of the trial's data, their proposed Clinical Data Management System (CDMS) will be used. In this case, e-Clinical will be used. e-Clinical is a web-based CDMS developed by Dynamic and following all safety law requirements described in the clinical investigation projects normative 21 CRF Part II.

Data will be then directly entered in the study database in electronic format following trial's Standard Operating Procedures (SOPs) in which study nurses will be trained. A Data Validation Plan (DVP) specifying all the Data Quality Checks (Data Queries) will be developed by the CRO to assure and control the quality of the data. These checks will be then programed into the eCRFs. Thus, data quality will be addressed at different levels. At the low level, data will be validated during data entry, i.e. when the study nurses are completing the eCRFs. Any error will be warned to the nurses then to be solved, if possible, during the completion of the eCRF. The intermediate level will be triggered once the data is sent to the server through the web application. At that point, a qualified data manager will verify that all the Data Quality Checks defined in the DVP

are met. At the highest level, during the proposed routine monitoring visits defined in the Monitoring Plan, the quality of a subset of the records produced during the study will be assessed and validated.

A Data Management Plan (DMP) and the Monitoring Plan will be prepared by the CRO describing in detail all the aspects related to the data management and monitoring of data.

Concomitant medications registered on the database will be coded using the WHO Drug Reference List, which employs the Anatomical Therapeutic Chemical classification system. Medical history/current medical conditions and adverse events will be coded using the Medical dictionary for regulatory activities (MedDRA) terminology.

### g. Quality assurance and quality control

Implementation of this study will be directed by the protocol as well as study specific SOPs. Quality assurance and quality control systems will be implemented and maintain with eSOPs to ensure that trials are conducted and data are generated, recorded and reported in compliance with the protocol, GCP, and the applicable regulatory requirement(s).

The eSOPs will outline procedures for conducting study visits, data and forms processing, AE assessment, management and reporting, dispensing study drugs and documenting drugs accountability, and other study operations.

Close cooperation between the sponsor, investigators, biostatisticians, data managers, and other study team members will be necessary in order to track study progress, respond to queries about proper study implementation, and address other issues in a timely manner. Assignment of all sponsor and investigator responsibilities for this project are specified in the project Consortium Agreement.

### h. Trial monitoring

The objectives of the trial monitoring are the following:

- To ensure that for all trial's records reviewed, the participants meet eligibility criteria, including a signed informed consent document
- To ensure that data entered into the electronic case report forms (eCRFs) are accurate and complete for all fields that will be reviewed
- To ensure that the on-site source documentation is consistent with the data entered into the eCRFs
- To ensure that regulatory documentation is present, complete and in compliance with GCPs, ICH and local regulations.
- To ensure that all study drugs are handled in accordance with investigational product procedures and accountability is accurately documented
- To ensure that the study is being conducted in compliance with the protocol, the SOPs, the GCP and local regulations.
- To ensure that site has written laboratory procedures, documentation of instrument calibration, and QA policies. A specimen audit will also be performed
- To ensure that site's trial and regulatory documents are in place, updated, and stored appropriately

The clinical monitoring will be performed by the CRO Dynamic Solutions. They will prepare the Monitoring Plan and will define the different types of monitoring visits.

### i. Data analysis

The data analysis plan will be developed by the CRO Dynamic Solutions in collaboration with all investigators. In general, it will include the following aspects:

#### Statistical analysis populations

The assignment of participants into analysis populations will be performed prior to database lock and any data analysis. The following populations are defined:

a) Intention to treat (ITT)

This population will include all randomized pregnant women and will be the target population for the efficacy analysis. Following the intention-to-treat principle, patients will be analyzed according to the treatment they were assigned to at randomization.

b) According to protocol (ATP)

This population will include all women who fulfil all the inclusion-exclusion criteria, took the 14 days HCQ treatment and from whom data is available for the analysis.

c) Safety

This population will include all patients who received at least one dose of HCQ/placebo and had at least a follow-up visit. The participant's safety will be analyzed by trial arm.

#### Patient demographic data and other baseline characteristics

All data will be listed and will consist of data collected/measured prior to randomization. Background and demographic characteristics of patients will be summarized for study arm.

Variables included in this analysis will be: maternal age, gestational age at recruitment (mean and by category), parity (mean and by category), etnic group, municipality, previous ANC visits before the recruitment, height (cm) and Hb results at first ANC visit.

#### Statistical models and methods of analysis

The primary analysis of the trial will be:

- The comparison of the proportion of negative SARS-CoV-2 PCR at day 14 after initiating treatment administration among those women who were SARS-CoV-2 PCR positive at recruitment in the ITT and ATP cohorts.
- The comparison of the proportion of pregnant women who were negative SARS-CoV-2 PCR at recruitment and close contacts of confirmed cases of SARS-CoV-2 infection, with a positive PCR for the infection at day 14, in the ITT and ATP cohorts.

Proportions will be compared between the two intervention-arms within each group using the Fisher exact test and presented as relative risk ratio (RR) or reduction of the RR (1- RR *100%) if RR lower than 1. Adjustment for co-variates and possible confounders will be done using Poisson regression with a log link and robust estimate of the covariance (Huber method), using the method proposed by Zou et al. 2014 and 2013.

Continuous variables will be compared between groups using Wilcoxon rank sum test and the effect presented as Mean Difference. Adjustment for co-variates and possible cofounders will be done using ordinary least square regression. Variables will be transformed to the logarithm scale if normality is improved and results presented as Proportional Difference. If after transformation, non-normality of the residuals is detected using diagnostic regression plots, robust intervals of confidence and Wald test will be presented instead.

## 6. Benefit/risk assessment

Infection with SARS-CoV-2 can be unpredictable in its severity, but a 3.4% mortality rate has been observed among clinical pneumonia cases. The elderly (>60 years) and those with medical co-morbidities are at highest risk of poor outcomes (29). Pregnant women are one of the vulnerable groups for this infection with increased severity of the disease if they become infected (3,12). The efficacy of HCQ in preventing the infection and reducing the severity of the disease is very limited and therefore, there is equipoise as to whether the *in vitro* efficacy of HCQ will translate into preventive efficacy from disease or even a reduction in viral shedding. On the other hand, the safety of HCQ in pregnancy is well established in pregnancy. Thus, the potential benefit-to-risk ratio for testing HCQ in pregnant women is favourable.

## 7. Ethical considerations

The project will be carried out with the highest standards of research integrity set out in the European Code of Conduct for Research Integrity and in total agreement with the applicable international, EU and Spanish national law.

Sampling of humans will always be made under The Nuremberg Code (1947) addressing volunteer consent and proper acting, the Revised Declaration of Helsinki and the convention for the protection of human rights and dignity of human beings with regard to the application of biology and medicine called the "Convention on Human Rights and Biomedicine" (Council of Europe, Oviedo, 1997) and its additional protocol on biomedical research (2005). All samples and databases will be anonymized.

In agreement with this, the ethical issues encountered are addressed in detail below.

### a. Human participants involved

The COVID-Preg study is designed to respond to the urgent questions related to finding effective preventive interventions to reduce the risk and consequences of of SARS-CoV-2 infection in pregnant women and their infants. This is needed for increasing the effectiveness of preventive tools and improving clinical management, allowing women to make informed health decisions. A fundamental ethical requirement in any clinical research is that the participants are those least vulnerable, in this case it would pregnant women; however, scientific evidence shows that efficacy and safety findings from COVID-19 control strategies evaluated in non- pregnant adults cannot be directly extrapolated to pregnant women. Pregnant women require specifically tailored interventions including those related to COVID-19 control.

Both oral and written communication will be used to provide information to pregnant women who are invited and interested in participating in the study. If a woman meeting inclusion criteria is interested in participating, she will be informed about the objectives and procedures of the study and about the risks and benefits of enrolling herself in the study. Ample time, adapted to each woman needs, will be given for consideration of the consent forms, and any questions will be responded by the investigator before the consent is signed.

### b. Informed consent

Informed consent is a process that is initiated prior to the individual's agreeing to participate in the study and continuing throughout the individual's study participation. The informed consent will be sent for approval to the Institutional Review Board (IRB) and the participants will be asked to read and review the document.

The consent form for the clinical trial (Annex 4) has been designed to provide information on the following aspects: general COVID-19 education, information about individual's rights as a study participant, what biological samples will be collected, the use of these samples, the

schedule of follow-up visits, confidentiality of personal data collected and potential risks and benefits of the study. It also specifies that participants have the right to leave the study whenever they decide and that this will not affect the clinical care that the patient will receive thereafter. The consent form is thorough but understandable by a lay person.

Three identical copies of the consent form will be signed. Two of the copies will be provided to the volunteer (one to be forwarded to the health system worker responsible for the control of her pregnancy) and the other copy will be kept in the study file by the site investigator.

### c. Inclusion of vulnerable populations

Vulnerable populations, such as pregnant women and their offspring will be participants in this investigation. This is justified by the nature of the project. The results of the investigation will be of relevance first and primarily for them.

To respond to the research questions both pregnant women and their infants need to be enrolled. Because we recognised that we will be working with particularly vulnerable populations, extreme care will be taken in all study aspects and in the process of informed consent in particular; this will be explained in a way that is culturally acceptable, understandable and avoiding any interpretation or intimidation. Before enrolment, written informed consent will be obtained from the parents or legal guardians of the minors (who will also be asked to give assent).

### d. Collection of biological samples

In order to evaluate the efficacy of the study drug, maternal and infant biological samples will be collected for the clinical trial. The samples required to perform these tests are harmless but can cause small discomfort especially in the newborn.

Results of the tests will be communicated to the clinician/nurse in charge and to the mother/parents. Adequate clinical management and treatment will be provided according to the test results.

As per International Good Clinical and Laboratory Practices, the origin of the samples collected will be kept, the necessary authorisation for samples collection will be obtained and sample's donors will be fully informed.

### e. Data protection

The management, communication and transfer of personal data of all participants will be in compliance with EU Regulation 2016/679 of the European Parliament and of the Council of April 27, 2016, which is mandatory since May 25, 2018, and with the Organic Law 3/2018, of December 5, on the Protection of Personal Data and digital rights guarantee. The collected data will be uniquely identified with a code, so no personal information will be collected that may allow identifying the participants. Only the study doctor and his/her collaborators with the right of access to the source data (medical history), will be able to relate the data collected in the study with the patient's medical history. A comprehensive section on data protection has been included in the Informed consent document.

### f. Ethical and regulatory approval

The trial will be conducted in accordance with Good Clinical Practice Guidelines, the Declaration of Helsinki, and national regulations (Royal Decree 1090/2015). The protocol and the informed consent forms will be submitted to the reference ethical committee of Hospital Clinic and to the Spanish Regulatory Agency (AEMPS), and the study will only start at each study site after having received approval from the specific ethical body of each site, if needed. No deviations from, or

changes to the protocol will be initiated without prior ethics committees' approval of any specific amendment.

## 8. Administrative aspects

### a. Collaboration agreements

Prior to the start of the trial, the COVID-Preg project collaboration agreement will be signed between all hospitals and ISGlobal.

### b. Insurance

A liability insurance has been taken and will cover all study participants.

## 9. Budget

This clinical trial has been funded by public funds through an open competitive call on COVID-19 disease and SARS-CoV-2 infection.

### a. Provision of study drugs

HCQ and placebo were donated by Laboratorios Rubió.

### b. In kind contributions of participating institutions

All study institutions, including the Sponsor, will contribute to the trial with in kind contributions of personnel time and internal resources.

## 10. Dissemination of results

A project communication plan will be developed in order to ensure timely, accurate, and effective communication among partners and key stakeholders. The plan will outline responsibilities for data management and sharing and communication with the external community, as well as a publication strategy. Agreement will be sought on how and to whom information will be shared to best meet the project and public awareness goals.

After concluding the trial analysis of all relevant data, results will be made available to all partners and key stakeholders. The project members will actively promote sharing and dissemination of results within the scientific community and beyond. The project members will disseminate information to the scientific community through reports, scientific publications in international journals, presentations at scientific forums.

## 11. Timeline

The trial recruitment will start after ethical and regulatory clearances are obtained.

Based on the number of deliveries at each study site, recruitment is expected to last 3-4 months, intervention during pregnancy and delivery will extend 9 additional months. Consequently, the estimated trial completion will be 12 months.

## 12. References


1. WHO. Coronavirus Disease (COVID-19) Pandemic. World Health Organization. 2020;Available at https://www.who.int/emergencies/diseases/novel-coronavirus-2019 [accessed April 2020].
2. The Report of the WHO-China Joint Mission on Coronavirus Disease 2019 (COVID-19). 16-24 February 2020.[Accessed 07/04/20. Available at: https://www.who.int/docs/default-source/coronaviruse/who-china-joint-mission-on-covid-19-final-report.pdf].
3. Chen H, Guo J, Wang C, Luo F, Yu X, Zhang W, et al. Clinical characteristics and intrauterine vertical transmission potential of COVID-19 infection in nine pregnant women: a retrospective review of medical records. Lancet (London, England). 2020 Mar;395(10226):809–15.
4. Liu H, Wang LL, Zhao SJ, Kwak-Kim J, Mor G, Liao AH. Why are pregnant women susceptible to COVID-19? An immunological viewpoint. J Reprod Immunol [Internet]. 2020;139(February):103122. Available from: https://doi.org/10.1016/j.jri.2020.103122
5. Visscher HC, Visscher RD. Indirect obstetric deaths in the state of Michigan 1960-1968. Am J Obstet Gynecol. 1971 Apr;109(8):1187–96.
6. Castillo P, Hurtado JC, Martinez MJ, Jordao D, Lovane L, Ismail MR, et al. Validity of a minimally invasive autopsy for cause of death determination in maternal deaths in Mozambique: An observational study. PLoS Med. 2017 Nov;14(11):e1002431.
7. Madinger NE, Greenspoon JS, Ellrodt AG. Pneumonia during pregnancy: has modern technology improved maternal and fetal outcome? Am J Obstet Gynecol. 1989 Sep;161(3):657–62.
8. Rigby FB, Pastorek JG 2nd. Pneumonia during pregnancy. Clin Obstet Gynecol. 1996 Mar;39(1):107–19.
9. Gottfredsson M. [The Spanish flu in Iceland 1918. Lessons in medicine and history]. Laeknabladid. 2008 Nov;94(11):737–45.
10. Jamieson DJ, Honein MA, Rasmussen SA, Williams JL, Swerdlow DL, Biggerstaff MS, et al. H1N1 2009 influenza virus infection during pregnancy in the USA. Lancet (London, England). 2009 Aug;374(9688):451–8.
11. Wong SF, Chow KM, Leung TN, Ng WF, Ng TK, Shek CC, et al. Pregnancy and perinatal outcomes of women with severe acute respiratory syndrome. Am J Obstet Gynecol. 2004 Jul;191(1):292–7.
12. Schwartz DA, Graham AL. Potential Maternal and Infant Outcomes from (Wuhan) Coronavirus 2019-nCoV Infecting Pregnant Women: Lessons from SARS, MERS, and Other Human Coronavirus Infections. Viruses. 2020 Feb;12(2).
13. World Health Organization. Clinical management of severe acute respiratory infection when novel coronavirus (nCoV) infection is suspected https://www.who.int/publications- detail/clinical-management-of-severe-acute-respiratory-infection-when-novel-coronav.
14. Vincent MJ, Bergeron E, Benjannet S, Erickson BR, Rollin PE, Ksiazek TG, et al. Chloroquine is a potent inhibitor of SARS coronavirus infection and spread. Virol J. 2005 Aug;2:69.
15. Yao X, Ye F, Zhang M, Cui C, Huang B, Niu P, et al. In Vitro Antiviral Activity and Projection of Optimized Dosing Design of Hydroxychloroquine for the Treatment of Severe Acute Respiratory Syndrome Coronavirus 2 (SARS-CoV-2). Clin Infect Dis. 2020 Mar;
16. Gao J, Tian Z, Yang X. Breakthrough: Chloroquine phosphate has shown apparent efficacy in treatment of COVID-19 associated pneumonia in clinical studies. Biosci Trends. 2020 Mar;14(1):72–3.
17. Gautret P, Lagier J-C, Parola P, Hoang VT, Meddeb L, Sevestre J, et al. Clinical and

microbiological effect of a combination of hydroxychloroquine and azithromycin in 80 COVID-19 patients with at least a six-day follow up: A pilot observational study. Travel Med Infect Dis. 2020 Apr;101663.
18. Sperber K, Hom C, Chao CP, Shapiro D, Ash J. Systematic review of hydroxychloroquine use in pregnant patients with autoimmune diseases. Pediatr Rheumatol Online J. 2009 May;7:9.
19. Nosten F, McGready R, d'Alessandro U, Bonell A, Verhoeff F, Menendez C, et al. Antimalarial drugs in pregnancy: a review. Curr Drug Saf. 2006 Jan;1(1):1–15.
20. Ministerio de Sanidad. Gobierno de España., III. I. Situación de COVID-19 en España [Actualizado a 19 de abril de 2020 a las 21:00 horas] Basada en la notificación diaria de casos agregados de COVID-19 al Ministerio de Sanidad. Available from: https://covid19.isciii.es/
21. Figueras F, Meler E, Iraola A, Eixarch E, Coll O, Figueras J, et al. Customized birthweight standards for a Spanish population. Eur J Obstet Gynecol Reprod Biol. 2008 Jan;136(1):20–4.
22. European Centre for Disease Prevention and Control. Contact tracing: public health management of persons, including healthcare workers, having had contact with COVID-19 cases in the European Union – second update, 8 April 2020. Stockholm: ECDC; 2020. Available from: https://www.ecdc.europa.eu/sites/default/files/documents/Contact-tracing-Public-health-management-persons-including-healthcare-workers-having-had-contact-with-COVID-19-cases-in-the-European-Union–second-update_0.pdf
23. Gueta I, Klempfner R, Markovits N, Halkin H, Segev S, Rott D, et al. Clinically significant incidental QTc prolongation is subject to within-individual variability. Ann Noninvasive Electrocardiol. 2020;25(2):1–7.
24. CDC. Public Health Guidance for Community-Level Preparedness and Response to Severe Acute Respiratory Syndrome (SARS) Version 2 Supplement F: Laboratory Guidance III. Diagnostic Assays [Internet]. Available from: https://www.cdc.gov/sars/guidance/f-lab/assays.html
25. Wu H-S, Chiu S-C, Tseng T-C, Lin S-F, Lin J-H, Hsu Y-H, et al. Serologic and molecular biologic methods for SARS-associated coronavirus infection, Taiwan. Emerg Infect Dis. 2004 Feb;10(2):304–10.
26. CDC. CDC 2019-Novel Coronavirus (2019-nCoV) Real-Time RT-PCR Diagnostic Panel Catalog # 2019-nCoVEUA-01 1000 reactions. 2020. p. CDC-006-00019, Revision: 03 03/30/2020.
27. Baud D, Greub G, Frave G, Gengler C, Jaton K, Dubruc E, et al. Second-Trimester Miscarriage in a Pregnant Woman With SARS-CoV-2 Infection. JAMA - J Am Med Assoc. 2020;1–3.
28. Mosha D, Mazuguni F, Mrema S, Abdulla S, Genton B. Medication exposure during pregnancy: a pilot pharmacovigilance system using health and demographic surveillance platform. BMC Pregnancy Childbirth. 2014 Sep;14:322.
29. Diseases TLI. Challenges of coronavirus disease 2019. Vol. 20, The Lancet. Infectious diseases. United States; 2020. p. 261.
